# Prevalence and associations of active trachoma among rural preschool children in Wadla district, northern Ethiopia

**DOI:** 10.1186/s12886-020-01585-9

**Published:** 2020-08-26

**Authors:** Mesfin Wudu Kassaw, Ayele Mamo Abebe, Kirubel Dagnaw Tegegne, Mikiyas Amare Getu, Woldemichael Tadesse Bihonegn

**Affiliations:** 1 Department of nursing, Woldia University, Woldia, Ethiopia; 2 Department of nursing, Debre Berhan University, Debre Berhan, Ethiopia; 3 Department of nursing, Wollo University, Dessie, Ethiopia; 4 Department of nursing, Samara University, Samara, Ethiopia

**Keywords:** Active trachoma, Associations, Water, Sanitation, Hygiene, Wadla district, Ethiopia

## Abstract

**Background:**

Trachoma is a neglected eye disease and an important cause of preventable corneal blindness. In endemic areas, initial infection can occur in early childhood and following a recurrent episodes, it progresses to scarring and visual impairment. Trachoma disappeared from high income countries through enhancements of hygiene and sanitation but the disease is still a challenge in developing countries. In Ethiopia, data indicate that Amhara is the region with the highest prevalence of active trachoma. The aim of this study was to assess the prevalence and associations of active trachoma among rural preschool children in Wadla district, Amhara region, Ethiopia.

**Methods:**

In this study, 596 children were screened for signs of active trachoma by using cluster-sampling technique. Following pre-testing of the survey instrument in a different district, questions about socio-demographic status were delivered for heads of households. Integrated eye care workers, previously trained to undertake trachoma screening for one month, performed eye examination. The logistic regression model was used to look for associations of active trachoma.

**Results:**

The prevalence of active trachoma among rural preschool children in Wadla district was 22%. Low economic status (adjusted odds ratio [AOR]3.8 (95%CI 1.3–11.4), being 37–48 months old (4.2;1.5–12.0), living in a house with thatched roof (4.4;1.4–13.6), presence of flies in a home (4.6;2.1–9.9), once-weekly face-washing frequency (8.6;2.5–29.3), having a face that had not been washed for longer than a week (10.6;2.9–37.7), and not using soap (4.5;1.8–11.3) had association to active trachoma.

**Conclusion:**

The prevalence of active trachoma among rural pre-school children in Wadla district was high. This indicates that Trachoma is still a public health problem in the district. This high prevalence calls for further interventions to prevent future trachomatis blindness.

## Background

Trachoma is a neglected eye disease and an important cause of preventable corneal blindness [1, 2], which is categorized into active and cicatricial types of trachoma [2, 3]. In endemic areas, cycles of infection with *Chlamydia trachomatis* progress to scarring, trachomatis trichiasis, and corneal opacity [4–6]. Trachoma is a disease of poverty and poor hygiene [7] that found primarily in children [4], with the late-stage disease more frequently seen in adult women than adult men, possibly because of women’s greater time spent in proximity to children. Pieces of literature indicate that preschool children are the main pool of ocular *C. trachomatis* infection [8, 9].Active trachoma can be an extremely common problem in children, with prevalence estimates of 60–90% [10]. Ocular C. trachomatis is believed to be transmitted through hand-to-hand contact, sharing of towels, fomites, pillows, and eye-seeking flies [11].

Globally, an estimated 2.5 million people had trachomatis trichiasis, needing surgery (S) to prevent ongoing visual impairment. Another nearly 142 million people lived in districts in which the prevalence of active trachoma met WHO-defined criteria for intervention with antibiotics (A) and interventions to promote facial cleanliness (F) and improve the environment (E), in order to prevent future trichiasis cases. Ethiopia is the most trachoma affected country: more than half of the 142 million people needing the A, F and E components of the “SAFE strategy” in 2019 lived here, Ethiopia [12]. Within Ethiopia, Amhara region has the highest trachoma burden [13], although, the prevalence and associations of active trachoma vary from setting to setting. Hence, studying the differences may help to tailor local control approaches. This is why we undertook investigations in Wadla district, Amhara region after 5 successive years of Zithromax administration in order to re-estimate the prevalence of active trachoma and examine its associations.

## Methods

### Study design, period and setting

A community-based cross-sectional study design was used. Fieldwork was undertaken from March 11, 2017 to April 26, 2107. The estimated population of Wadla district was 128,170 with 64,574 males and 63,596 females. There were 28,414 households in this district with an average of 4.5 persons per house. The district had 1 general hospital, 7 health centers, and 20 health posts.

### Population

The sampling frame was children aged 1 to 5 years old in 150 rural villages of Wadla district. The study units were heads from the selected rural households that also had preschool children.

### Sample size determination

We estimated the required sample size using the single population proportion formula. We assumed, based on previous surveys, an observed prevalence of active trachoma (12.1%) [14], which we wished to estimate with 95% confidence within ±5%. We used a design effect of 1.5, and allowed for 10% non- response rate. Through multiplying the sample size by the design effect, 1.5 and incorporating a 10% non-response rate, we estimated273 children that were needed to be framed in selected households.

### Sampling technique

**Fig. 1:**
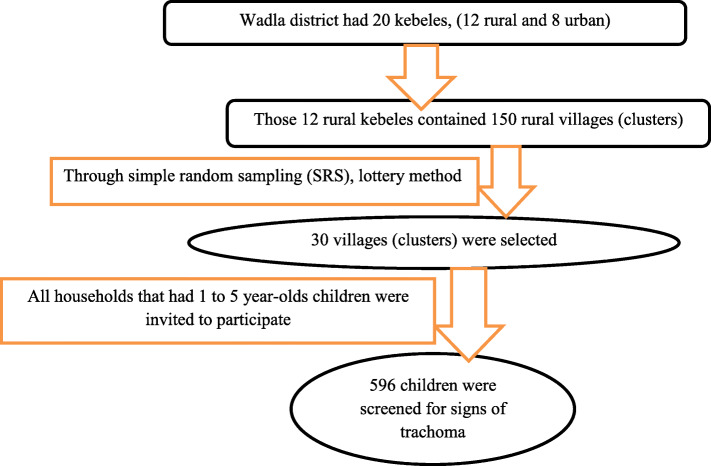
: The schematic diagram of sampling procedure in selecting preschool children from rural Wadla district, northern Ethiopia, 2017. The sample size calculated was 273 using single population proportion formula, but as the sampling procedure was cluster sampling, the numbers of screened children were 596 from all 30 villages.

A multistage cluster sampling technique was applied. Wadla district had 20 kebeles (sub-districts) that comprise 247 villages. Twelve of the kebeles were rural, whereas eight of the kebeles were urban. Regarding the villages, 150 of the 247 villages were rural. We used simple random sampling to select 30 of the 150 rural villages. There were 967 households in the selected 30 villages, but only 499 of those households had preschool children. Thus, those 499 households were visited. Heads of households were interviewed for socio-demographic and economic information, plus housing and environmental conditions, and all children aged between 1 and 5 years who had been resident in the district for at least six months were invited to be examined. Eye examiners used the WHO simplified trachoma grading scheme to grade signs of trachoma [15] (Fig. 1).

### Definitions

#### Clean face

A face of child that was free of eye discharges, nose discharges or flies at the time of eye examination.

#### Preschool

Children whose age were greater than and equal to 1 year and less than or equal to 5 years old.

#### Village

A grouping of homes that contained at least 30 households organized as one peasant association.

#### Fly in a home

When there is/are a countable fly in a house during data collection, despite the number of flies.

#### Active trachoma

The presence of at least one of the two signs of active trachoma according to the WHO simplified trachoma grading scheme (TF or TI) in at least one eye [16].

#### Trachomatis inflammation—follicular (TF)

The presence of five or more follicles each having a diameter of at least 0.5 mm in the central part of the upper tarsal conjunctiva [16].

#### Trachomatis inflammation—intense (TI)

A pronounced inflammatory thickening of the upper tarsal conjunctiva that obscures more than half of the normal deep tarsal blood vessels [16].

#### Trachomatis scarring (TS)

The presence of easily visible scarring in the upper tarsal conjunctiva [16].

#### Trachomatis trichiasis (TT)

The presence of at least one eyelash rubs on the eyeball or evidence of removal of in-turned eyelashes in the two weeks before examination [16].

#### Corneal opacity (CO)

the presence of easily visible corneal opacity over the pupil [16].

### Exclusion and inclusion criteria

All the children belong to the appropriate age range mentioned above and who had lived in the district for at least 6 months, who were resident in selected villages and available at the time of study were invited to be included. Children who were seriously ill or for whom informed consent was not given by parents or guardians were excluded.

### Measurements

The outcome variable was active trachoma and measured by physical examination. A number of dependent variables were considered that includes socio-demographic, environmental, hygiene and sanitation, and children’s demographic data.

### Data collection tools and procedures

In collecting the data, face to face interviews, observation using a checklist and clinical eye examination were used. Experienced health informatics professionals were using structured interview questions that prepared from pieces of literature ( [17, 18], and Additional file 1), while they collected the data on a socio-demographic status, environmental, and housing conditions. All the questionnaires of socio-demographic status, housing, and environmental condition, observation checklist, and eye examination tools were pretested and validated before data collection in Kosomender, Meket district, a district bordering Wadla to the north. A household wealth index was developed using composite indicators for rural residents’ assets: livestock ownership, size of agricultural land and quantity of crop production.

Two integrated eye care workers performed the eye examination. Those integrated eye care workers are ophthalmic nurses who had been previously trained for a total duration of one month for the purposes of contributing to the 2013–2014national trachoma survey. The Carter Center delivered that previous training using both pictures and live patients as media of instruction. However, for the purpose of this study, the trachoma graders undertook refreshment training for 5 days. This training considers examination of58 live patients and 100 pictures of different trachoma signs. Trainers, whose grades were used as the gold-standard assessment assessed graders. The training was also delivered for interviewers. Interviewers assisted graders by recording clinical grades, and data related to each household’s socio-demographic status and environmental situation. The trainers emphasized on the objectives, procedures of data collection and mode of communication between graders and interviewers. When undertaking the fieldwork, graders initially observed the eyelashes and cornea of study subjects, looking for TT and CO, then everted the upper lid and inspected the upper tarsal conjunctiva for TF, TI, or TS. Binocular lenses (× 2.5) and penlight torches were used [4] to magnify the examined eye.

### Data analysis and presentation

**Fig. 2:**
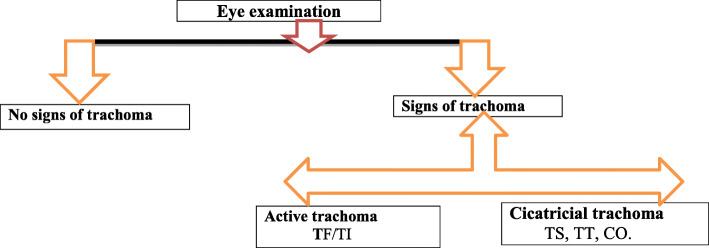
**:** The schematic presentation of eye examination outcome and result reporting procedure of active trachoma among rural preschool children in Wadla district, northern Ethiopia, 2017.

The data were checked for completeness, coded and entered into Epi-info version 7, and transferred to SPSS version 23 for analysis. The data were checked for normality using Hosmer-Lemeshow-goodness-of-fit. A univariate analysis model were carried out, and variables that had a *p*-value of < 0.25 in a binary logistic regression model were included to the multivariate logistic regression analysis. Potential co-linearity was also considered and tested using multi co-linearity model in considering tolerance and variance inflection factor (VIF). Variables with a *p*-value of < 0.05 in the multivariate logistic regression analysis were considered as statistically significant. A principal component analysis was performed to categorize households’ wealth into poorer, poorest, middle, richer, and richest. However, for the presentation of the variables, the wealth index was grouped into three; lowest, middle, and highest. The procedure of eye examination and result reporting presented in Fig. 2. Both active trachoma and cicatricial trachoma were modeled as outcome variables. Thus, children were screened for both Active and cicatricial types of trachoma (Fig. 2).

### Data quality assurance

The questionnaire was prepared in English and translated to Amharic, then re-translated to English (to check for accuracy) by individuals, who are fluent in both English and Amharic. Both graders and one of the researchers, principal investigator had been participated in a community-based trachoma survey and training before starting the present study. The interviewers had also previous experience in a community-based data collection.

## Results

In the study villages, there were 610 preschool children from 499 households. However, only 596 preschool children were examined and gave a response rate of 100%. The remaining 14 children were not involved in the screening phase because of the exclusion criteria and absenteeism after repeated household visit. More than three-fourths 383(77%) of households had male heads. The range in the number of residents per household was 2 to 10 with a median of five. The range in the number of 1 to 5 years old children per household was 1 to 3 with a median of one. All the 499 families were Amhara in ethnicity and followed Ethiopian orthodox Christianity, and 325 (65%) fathers, and 380 (76%) mothers were unable to read and write. Four hundred and sixty-six (93%) fathers were farmers and 16 (3%) fathers were government employees (Table 1).

**Table 1:** Socio-demographic characteristics of heads of households in rural Wadla district, northern Ethiopia, 2017

Variables	Frequency (*n* = 499)	Percent (%)
Sex of head of a household		
Male	383	76.8
Female	116	23.2
Marital status of head of a household		
Married	492	98.6
Divorce	7	1.4
Wealth index		
Poor	144	28.9
Medium	279	55.9
Rich	76	15.2
Occupation of head of a household		
Farmer	466	93.4
Merchant	17	3.4
Government employee	16	3.2
Educational status of head of a household		
Unable to read and write	325	65.1
Able to read and write	109	21.8
Up to grade 8	35	7
Grade 9 to 12	19	3.8
Diploma and above	11	2.2
Educational status of mothers		
Unable to read and write	380	76.2
Able to read and write	55	11
Up to grade 8	23	4.6
Grade 9 to 12	35	7
Diploma	6	1.2
Number of rooms in the house (observation)		
One	424	85
Two and more	75	15
Family size		
Less than 6	286	57.3
Greater than and equal to 6	213	42.7
Total number of children less than five years in a house		
One	424	85
Two	69	13.8
Three	6	1.2
Number of children less than ten years in a house		
One	132	26.5
Two	240	48.1
Three	102	20.4
Four	25	5
Adult face washing habit (self-report)		
At least one times per a day	417	83.6
Less than 7 times per a week	82	16.4

In addition to the socio-demographic characteristics, the environmental characteristics of households are shown in Table 2.

**Table 2:** Environmental conditions of the study households in rural Wadla district, northern Ethiopia, 2017

Variables	Frequency (*n* = 499)	Percent (%)
Presence of fly in or around a house (observation)		
Present	242	48.5
Absent	257	51.5
Source of water (self-report)		
River	30	6.0
Unprotected well	12	2.4
Protected well	56	11.2
Pipe	401	80.4
Amount of water in a litter (self -report)		
Less than 20	180	36.1
20–40	162	32.5
40–60	92	18.4
60–80	49	9.8
Greater than 80	16	3.2
Total time taken to reach to water source (self-report)		
Less than and equal to 1/2 h.	459	92
Greater than 1/2 h.	40	8
Place of cooking (observation)		
In the same room of living house	157	31.7
In the same house but in a kitchen	166	33.3
A kitchen constructed against outside wall of the house	3	.6
Isolated kitchen	173	34.7
Presence of window in a kitchen (observation)		
Yes	248	49.7
No	251	50.3
Household waste removal (self-report)		
Burn it	312	62.5
Bury it	90	18
Dispose in the farm	93	18.8
Dispose in another place	4	.8
Presence of latrine (observation)		
Present	371	74.3
Absent	128	25.7
Presence of feces at open field in nearby a house (observation)		
Present	243	48.7
Absent	256	51.3
Presence of cattle in a household (observation)		
Present	439	87.9
Absent	60	12.1
Cattle sheltering (*n* = 439) (observation)		
In the same room where family lives	128	29.1
In the same living house but in a separate room	203	46.2
Attached shelter against outside of the house	6	1.6
Isolated shelter far from the house	102	23.1

Among children examined for signs of active trachoma, 301 (51%) were males, and 295 (49%) were females. The median age of children was 36 months **(**Table 3**).**

### Factors associated with active trachoma

On binary logistic regression analysis, lowest economic status, being in the age group of 24–36 months old, unable to read and write educational status of fathers, unable to read and write educational status of mothers, living in a house with a thatched grass roof, fly in a house, and a MUAC of children < 13.9 cm associated with active trachoma (Table 4). However, on the multivariable logistic regression analysis, only lowest economic status (AOR (95% CI), (3.80 (1.27–11.42)), being 37–48 months old (4.21 (1.47–12.03)), living in a house with a thatched grass roof (4.40 (1.42–13.59)), or presence of fly in a home (4.6 (2.1–9.9)) were increasing the odds of active trachoma **(**Table 4**).**

**Table 3:** Socio-demographic characteristics of the pre-school children in rural Wadla district, northern Ethiopia, 2017

Variables	Frequency (*n* = 596)	Percent
Sex of children		
Male	301	50.5
Female	295	49.5
Age of children in months (kebele registration book)		
12–24	208	34.9
25–36	102	17.10
37–48	129	21.6
49–59	157	26.3
Current breast-feeding status of children		
Yes	239	40.1
No	357	59.9
Face washing frequency of children (self-report)		
2 or more times per a day	108	18.1
Once daily	79	13.3
2 to 6 times per week	149	25
Once weekly	167	28
Stays unwashed for longer than a week.	93	15.6
Habit of child bathing for at least one times per a week (self-report)		
Yes	445	74.7
No	151	25.3
Use of soap for face washing(self-report)		
Yes	264	44.3
No	332	55.7
Use of soap for hand washing(self-report)		
Yes	254	42.6
No	342	57.4
Face of children on observation (observation)		
Clean face	280	47
Ocular discharge	89	14.9
Nasal discharge	75	12.6
Flies on the face of child		10.6
Ocular and nasal discharge	34	5.7
Ocular and nasal discharge and flies on the face	55	9.2
Presence of another eye problem(self-report)		
Yes	146	24.5
No	450	75.5
Type of eye problem (*n* = 146)		
Discharge	96	65.6
Itching	8	5.3
Excessive tear	25	17.1
Redness of eye	18	12.2
Took drug during mass drug administration in the last year(self-report)		
Yes	515	86.4
No	81	13.6

Of the 596 screened children for signs of trachoma, 56.2% of female children had trachoma. One hundred and thirty children had active trachoma, giving a prevalence of 22% [95%CI, 18–25%)]. One hundred and six children had TF, 13 had TI, and 11 had both TF and TI. There were no signs of TS, TT or CO. Two hundred and eighty (47%) children had clean face, 89 (15%) had ocular discharge, 75 (13%) had nasal discharge, 34 (6%) had both ocular and nasal discharge and 55 (9%) children had nasal discharge, ocular discharge, and fly on their face.

**Table 4:** Association of active trachoma and risk factors among pre-school children in rural Wadla district, northern Ethiopia, 2017

Variables	Trachoma (*n* = 596)		OR (95% CI)	
	Presence (%)	Absence (%)	COR	AOR
Type of house roof (observation)				
Clean iron	15 (11.5)	82 (17.6)	1.00	1.00
Thatch iron	24 (18.5)	141 (30.3)	0.9(0.5–1.9)	0.9 (0.3–2.8)
Clean grass	27 (20.8)	144 (30.9)	1.0(0.5–2.0)	0.7 (0.2–2.2)
Thatch grass	64 (49.2)	99 (21.2)	3.5 (1.9–6.7) *	4.4 (1.4–13.6) *
Fly in a house or in nearby (observation)				
Yes	96(73.8)	206 (44.2)	3.6 (2.3–5.5)	4.6 (2.1–9.9) *
No	34 (26.2)	260 (55.8)	1.00	1.00
Face washing frequency (self-report)				
Two and more times	9 (6.9)	99 (21.2)	1.00	1.00
Once daily	2 (1.5)	77 (16.5)	0.3 (0.1–1.4)	0.2 (0.03–1.3)
2 to 6 times per a week	15 (11.5)	134 (28.8)	1.2 (0.5–2.9)	1.366 (.365–5.114)
Once weekly	63 (48.5)	104 (22.3)	6.7 (3.1–14.1) *	8.7 (2.6–29.3) *
Unwashed for a week	41(31.5)	52 (11.2)	8.7 (3.9–19.2) *	10.6 (2.9–37.7) *
Soap for face washing(self-report)				
Used	26 (20)	238 (51.1)	1.00	1.00
Not used	104 (80)	228 (48.9)	4.2 (2.6–6.7) *>	4.5 (1.8–11.3) *
Soap for hand washing(self-report)				
Used	35 (26.9)	219 (47.0)	1.00	1.00
Not used	95(73.1)	247 (53.0)	2.4 (1.6–3.7) *	1.6 (0.8–3.6)
Household latrine (observation)				
Present	7 (21.2)	364 (78.1)	1.00	1.00
Absent	26 (78.8)	102 (21.9)	2.0 (1.3–3.0) *	5.0 (2.0–12.9) *
Household waste around the house (observation)				
Exist	80(61.5)	214 (45.9)	1.9 (1.3–2.8) *	3.4 (1.6–7.6) *
Not exist	50 (38.5)	252 (54.1)	1.00	1.00
Mothers educational status				
Unable to read and write	111 (85.4)	348 (74.7)	2.9 (1.3–6.6) *	0.8 (0.2–3.2)
Able to read and write	12 (9.2)	53 (11.4)	2.1 (0.8–5.7)	0.3 (0.1–1.6)
Attend formal education	7 (5.4)	65 (13.9)	1.00	1.00
Wealth index				
Poor	73 (56.2%)	101 (21.7)	4.6 (2.3–9.1) *	4.2 (1.5–12.0)
Medium	45 (34.6%)	288 (61.8)	1.003 (.506–1.988)	0.5(0.2–1.4)
Rich	12 (9.2%)	77(16.5)	1.000	1.00
MUAC of children				
Less than 13.9	81(62.3)	230 (49.4)	1.7 (1.1–2.52) *	1.3 (0.6–2.6)
Greater than 14	49(37.7)	236 (50.6)	1.00	1.00
Age of children (in months)				
12–24	42 (32.3)	166 (35.6)	0.8 (0.5–1.3)	0.7 (0.3–1.8)
25–36	14 (10.8)	88 (18.9)	0.5 (0.3–0.9) *	0.7(0.2–2.1)
37–48	36 (27.7)	93 (20)	1.2 (0.7–2.1)	2.7(.1.0–7.2)
49–59	38 (29.2)	119 (25.5)	1,00	1.00
Fathers education				
Unable to read and write	93 (71.5)	299 (64.2)	2.3 (1.1–4.7) *	1.4 (0.3–6.2)
Able to read and write	28 (21.5)	102 (21.9)	1.9(0.9–4.5)	2.1 (0.5–9.7)
Formal education	9 (6.9)	65 (13.9)	1.00	1.00

* = *p* < 0.05

## Discussion

The objective of this study was to assess the current prevalence of active trachoma and to identify its associations among children aged 1 to 5 years old in rural communities of Wadla district. The prevalence of active trachoma in this age group was 22%, [95%CI, 18–25%], whereas the prevalence of TF was 21%. Although the usual indicator age group for determining the need or otherwise for the A, F and E components of the SAFE strategy is the prevalence of TF in 1 to 9 years-old children, the prevalence that we estimate here suggests that three further years of antibiotic mass drug administration is likely to be required, according to WHO recommendation [19]. However, a study from northern Ethiopia reported that azithromycin mass treatment coverage in 2012 was 92.9% [20]. That reported mass azithromycin coverage was greater than the minimum coverage set by WHO, 80% [21]. The prevalence agreed with a review that indicated 70 million people in Ethiopia required MDA. This was the largest need of any other country in the world [22]. The prevalence of TI among 1 to 5 years old children here was 3.4%. Severe inflammatory trachoma is a risk factor for later cicatricial disease, particularly when the sign is observed repeatedly over a time [23]. In our subjects, reportedly face washing once weekly and having a face that had remained unwashed for longer than a week were associated with active trachoma. Similar associations had been seen elsewhere [18, 24]. We also found that the absence of a toilet or presence of human excreta near to a home increased the odds of there being active trachoma. Recent multi-country observational data support the link between inadequate access to sanitation and the likelihood of active trachoma [25]. In general, the associations that we found agreed with the previous published literature that suggests a strong links between trachoma and environmental factors related to water, sanitation, and hygiene. Some of these associations implicate the fly *Muscasorbens*, which oviposit in human excreta left exposed on the soil, as an important vector [26–28]. In this study, grassed and thatched house roof (AOR (95% CI), 4.402 (1.425–13.597) were increasing the odds of active trachoma. This association evidenced from central Ethiopia [29].In this study, not using soap was increasing the odds of active trachoma [(AOR (95%CI), 4.49 (1.79–11.29)]. This agreed with studies that were conducted in Dessie city and Gonder, Ethiopia [13, 30]. Unfortunately, we did not have any entomological data for this site. Other limitations of our analyses include our reliance on self-report for many of the exposure variables, and the exclusion of children aged 6 to 9 years old. However, this research estimates the prevalence of active trachoma among preschool children from rural area, and its associations, for the attention of policymakers interested in trachoma elimination in Wadala district, Amhara region, Ethiopia.

## Conclusions

The prevalence of active trachoma among rural preschool children in Wadla district was high, suggesting that active trachoma is still a public health problem in Wadla district. Environmental factors were found to be associated with active trachoma. This might suggest an ongoing need for implementation of the F and E components of the SAFE strategy for trachoma elimination in this district to prevent future trachomatis blindness.

## Supplementary information

**Additional file 1.**

## Data Availability

The data generated in this study will be available to researchers wishing to use the data for non-commercial purposes by asking the principal investigator Mr., Mesfin Wudu through his e-mail, mesfine12a@gmail.com.

## Abbreviations

COR: Crude odds ratio
AOR: Adjusted odds ratio
CI: Confidence interval
AT: Active Trachoma
SAFE: Surgery, Antibiotics, Facial cleanliness, Environmental improvement
GET2020: Global elimination of Trachoma by 2020
WHO: World Health Organization
TT: Trachomatis trichiasis
TF: Trachomatis inflammation—follicular
TI: Trachomatis inflammation—intense
TT: Trachomatis trichiasis
CO: Corneal opacity
MUAC: Mid upper arm circumference
MDA: Mass drug administration
