# Supplementary material for: Prevalence and associations of active trachoma among rural preschool children in Wadla district, northern Ethiopia
Source: BMC Ophthalmol. 2020 Aug 26;20:346. doi: 10.1186/s12886-020-01585-9 (PMC7449018; doi:10.1186/s12886-020-01585-9)
Supplement: Supplementary file 1 — Additional file 1. [file 12886_2020_1585_MOESM1_ESM.docx]

# Supplementary file

# Figure1

**Part. I. Questions to be answered at the household level**

The following questions (from 101-117) focus on the selected households and shall be responded by the head of the households

| No | Questions | Coding Categories | Skip |
| --- | --- | --- | --- |
| 101 | What is the sex of the head of the household? | 1. Male 2. Female |  |
| 102 | What is the religion of the head of the household? | 1.Orthodox 2.Protestant  3. Catholic 4.Muslim  5. Others (……….) |  |
| 103 | What is the marital status of the head of the household? | 1. Married 2.Single  3. Divorce 4. Other (……) |  |
| 104 | What is the ethnicity of the head of the household? | 1. Amhara 2. Tigray  3. Oromo 4. Other (….) |  |
| 105 | What is the occupation of head of the household? | 1. Farmer 2. Craftsmen  3. Merchant 4. Housewife  5. Pensioner 6.Other (…..)  7. Government employee |  |
| 106 | What is the educational status of head of the household? | 1. unable to write & read  2.Able to write & read  3.Attended formal school  4. Other (………….) |  |
| 107 | What is the condition of house roof? | 1.Thin & clean 2.Grass & clean  3. Thin& thatch 4. Grass & thatch |  |
| 108 | What is the family size? |  |  |
| 109 | What is number of children less than 10 years old in your home? | __________________ |  |
| 110 | What is the number of children less than 5 years old in your home? | __________________ |  |
| 111 | What is the number of rooms in the living house? |  |  |
| 112 | How are the fly densities in and around the compound? | 1. 1. Very few 2. Few (6 - 10) 2. 3. Medium in number (11- 15) 3. 4.Many (16 - 20) 5.Quite many (>20) |  |
| 113 | How many km far your home from the town? |  |  |
| 114 | How many km far your home from health center? |  |  |
| 115 | From where mainly do your family members get water for domestic use?(you can indicate >one option) | 1.River 2.Unprotected spring  3.Pond 4.Unprotected well  5. Rain water 6.Protected spring  7. Protect well 8. Pipe  9. Other (……………) |  |
| 116 | How much water do the family members consume per day for cooking, washing utensils and cloths and personal hygiene (in Liters)? | 1. <one pot (20 L) 2. 1 to 2 pots (20-40)  3. 2 to 3 pots (40-60) 4. 3 to 4 (60 - 80)  5. ≥ 4 pots (80) |  |
| 117 | For how long do you travel to get water for domestic use? (if two or more way, choice > one options) | 1. 1. Less than half an hour 2. 2. From 1/2hr. to 2hr. walk 3. 3. From 2hsr to 4hrs walk 4. 4. Longer than 4hrs walk. |  |

**Part II: The following questions (201 - 210)** are concerning the practice of the community in relation to trachoma

| No | Questions | Coding categories | Skip |
| --- | --- | --- | --- |
| 201 | Where do the family members cook? | 1. In the same room with live  2. In the same house with live but in a separate room  3. In separately constructed kitchen  4. In a kitchen constructed against the outside living house  5. If other (…………..) |  |
| 202 | Does the cooking room have window? | 1. Yes 2. No |  |
| 203 | What do you do to your domestically produced refuse (garbage)? | 1. Burn it 2. Bury it  3. Dispose in the farm  4. Simply dispose it in other place |  |
| 204 | If your answer to question 203 is option 4, where do you dispose it? | 1. Nearby living house  2. Far away from the living house  3. In the river or stream |  |
| 205 | Do you have latrine? | 1. Yes 2. No |  |
| 206 | Do you have own cattle? | 1. Yes 2.No |  |
| 207 | If your answer to Q 206 is yes, where do the cattle pass the night? | 1. In the same room where family lives.  2. In the same living house the family  lives but in a separate room  3. In a shelter constructed for them  around the house  4. Other (………) |  |
| 208 | Is there frequent adult face washing habit in your family? | 1. 1. Yes 2. No |  |
| 209 | Were you participating actively on community involvement about trachoma? | 1. 1. Yes 2. No |  |
| 210 | Did you take any health education about trachoma? | 1. 1. Yes 2. No |  |

**Part III. Questions about the child selected for the study**

The following questions (301 -316) focus on individual child selected for screening

| No | Questions | Coding Categories |
| --- | --- | --- |
| 301 | What is the age of the selected child in years? | __________________ |
| 302 | What is the sex of the selected child? | 1.Male 2.Female |
| 303 | Is the child feeds breast milk? | 1. Yes 2. No |
| 304 | What is the MUAC of the child? |  |
| 305 | How often does the selected child wash his/her face? | 1. 2 or more times /day.  2. Once daily  3. 2- 6 times per week  4. Once weekly  5. Stays unwashed for longer than a week. |
| 306 | Is there a habit of bathing children frequently? | 1. Yes 2. No |
| 307 | What is the case of the child during facial observation? | 1. Ocular discharge  2. Nasal discharge  3. Either ocular discharge or nasal discharge  4. Fly on the child’s face  5. Either ocular discharge, nasal discharge or fly on the child’s face |
| 308 | Does the selected child use soap when washing his/her face? | 1. Yes 2. No |
| 309 | Does the selected child use soap when washing his/her hand? | 1. Yes 2. No |
| 310 | Does the child has eye problem? | 1. Yes 2. No |
| 311 | If yes to Q 310, which is the case of the child | 1. Discharge 2. Itching  3.Excessive tear 4.Redness of eyes  5.Visual impairment 6.Photophobia  7.Swelling of eye lids 8.Blindness |
| 312 | Was the child taking a drug during MDA? | 1. 1. Yes 2. No |
| 313 | Result of the Eye examination for trachoma | 1. Yes 2. No |
| 314 | If Q 313 is yes, specify the stage of Trachoma |  |
| 315 | Presence of trachoma symptom in other family member | 1. Yes 2. No |
| 316 | If yes to Q 313, What is the age of that patient? | ___________________ |
